# Cost implications of treatment non-completion in a forensic personality disorder service

**DOI:** 10.1002/cbm.1866

**Published:** 2013-07-23

**Authors:** Christopher James Sampson, Marilyn James, Nick Huband, Steve Geelan, Mary McMurran

**Affiliations:** 1 School of Sociology and Social Policy, University of NottinghamNottingham, UK; 2 NIHR-CLAHRC-NDLNottingham, UK; 3 Nottinghamshire Healthcare NHS TrustNottingham, UK; 4 Institute of Mental Health, University of NottinghamNottingham, UK

## Abstract

**Background**A high proportion of individuals admitted to specialist secure hospital services for treatment of personality disorder do not complete treatment. Non-completion has been associated with poorer treatment outcomes and increased rates of recidivism and hospital readmission, when compared with individuals who do complete treatment or who do not receive treatment at all.

**Aims**In this study, we sought to determine the economic consequences of non-completion of treatment, using case study data from a secure hospital sample. Both health and criminal justice service perspectives were taken into account.

**Methods**Data were collected from a medium secure hospital personality disorder unit. A probabilistic decision-analytic model was constructed, using a Markov cohort simulation with 10,000 iterations. The expected cost differential between those who do and those who do not complete treatment was estimated, as was the probability of a cost differential over a 10-year post-admission time horizon.

**Results**On average, in the first 10 years following admission, those who do not complete treatment go on to incur £52,000 more in costs to the National Health Service and criminal justice system than those who complete treatment. The model estimates that the probability that non-completers incur greater costs than completers is 78%.

**Conclusion**It is possible that an improvement in treatment completion rates in secure hospital personality disorder units would lead to some cost savings. This might be achievable through better selection into treatment or improved strategies for engagement and retention. Our study highlights a financial cost to society of individuals discharged from secure hospital care when incompletely treated. We suggest that it could, therefore, be useful for secure hospitals to introduce routine monitoring of treatment completion. © 2013 The Authors. Criminal Behaviour and Mental Health published by John Wiley & Sons, Ltd.

Personality disorders affect many people, with an estimated 2.5 million cases in England, and a contribution to healthcare costs of around £8bn each year (McCrone et al., 2008). People diagnosed with personality disorders have a significantly impaired quality of life (Soeteman et al., 2008). Treatment can mitigate problems and reduce risk (Bateman and Fonagy, 2000; Leichsenring and Leibing, 2003), but treatment non-completion is common among those in treatment for personality disorder. In a recent meta-analysis, Swift and Greenberg (2012) found that psychotherapy non-completion averaged around 20% for all patients, with the highest rates for people with personality disorder (26%). Rates of non-completion of treatments specifically for personality disorders have been found to vary, averaging around 37% (McMurran et al., 2010). The high cost incurred by these service users warrants an economic analysis of the consequences of non-completion. Non-completion of treatment of personality disorder has been associated with a number of negative consequences, including retention of criminal attitudes (Cullen et al., 2012), poor global functioning and higher rates of hospitalisation (Karterud et al., 2003). Unfortunately, however, there is a lack of consensus on a definition of treatment completion, with some definitions based on clinical judgement and some upon duration of treatment. One important possible consequence of treatment non-completion is that this may lead to greater costs to society. Patients who do not complete treatment for borderline personality disorder, for example, have been found to spend three times longer in hospital than treatment completers (Webb and McMurran, 2009). Non-completers of an inpatient treatment programme for legally detained personality disordered offenders committed more offences in a 5-year follow-up period than did completers (McCarthy and Duggan, 2010). Furthermore, non-completers of general offender treatment programmes demonstrate higher rates of recidivism than individuals who do not receive treatment at all (McMurran and Theodosi, 2007).

The primary objective of this study was to determine the likelihood that, and extent to which, personality disordered offenders who complete treatment – ‘treatment completers’ – and those who do not complete treatment – ‘non-completers’ – go on to incur different costs to society following discharge. A secondary objective was to demonstrate the feasibility of using decision-analytic modelling techniques in the evaluation of personality disorder services.

## Method

### Decision modelling

Prospective trials of inpatient care for forensic mental health patients are rare. As such, it is seldom possible to carry out an economic evaluation of these services. When it is not possible to analyse trial data, methods of decision modelling represent a viable alternative. Decision-analytic modelling is a technique that employs both mathematical and statistical methods to simulate real life events (Briggs et al., 2006). Decision models enable decision makers to form policies under conditions of uncertainty in a real world context. Models present us with probabilities that a given event will occur under a set of pre-defined conditions. Events are associated with costs and benefits, enabling us to calculate expected costs and benefits of different interventions, the intervention with the greatest expected net benefit being the optimal choice. An important benefit of decision-analytic modelling is that, by making all parameters probabilistic, it allows for uncertainty around all inputs to the model and all decisions. This provides us with information regarding the level of uncertainty in our data, without needing to make arbitrary distinctions. Furthermore, modelling facilitates complete transparency in the assumptions made. There is good scope for economic modelling to be used in the evaluation of personality disorder treatments. Modelling techniques, such as Markov modelling, are used in many areas of health service evaluation, including chronic conditions with complex long-term outcomes such as rheumatoid arthritis (Chen et al., 2006) and diabetes (National Institute for Health and Clinical Excellence, 2008). Such modelling methods have, however, rarely been used in the evaluation of interventions for personality disorders. A recent study by Barrett and Byford (2012) used Markov modelling to evaluate the dangerous severe personality disorder programme, and there has been one other economic model developed to evaluate the cost-effectiveness of psychotherapy for personality disorder (Soeteman et al., 2010, 2011).

A model-based analysis of treatment non-completion has not previously been tried, nor has one related to the economic consequences of personality disorder more generally. This means that there are no existing models upon which to build our analysis. In our analysis, we use the technique of Markov modelling (Sonnenberg and Beck, 1993). Markov modelling has become popular in the evaluation of physical conditions. The operation of a Markov model is dependent on the condition that subjects can be defined as existing in one of a finite number of mutually exclusive and collectively exhaustive ‘states’. Subjects can then make transitions between these states based upon probabilities, with transitions occurring at the beginning of each Markov cycle. Each cycle spent in any one state is then associated with costs and outcomes, which can either be fixed or sampled from a probability distribution. Costs and outcomes are then accumulated over time. Markov models have a number of advantages over simpler methods in that they allow for a wider set of consequences and more realistic temporal dynamics.

**Figure 1:**
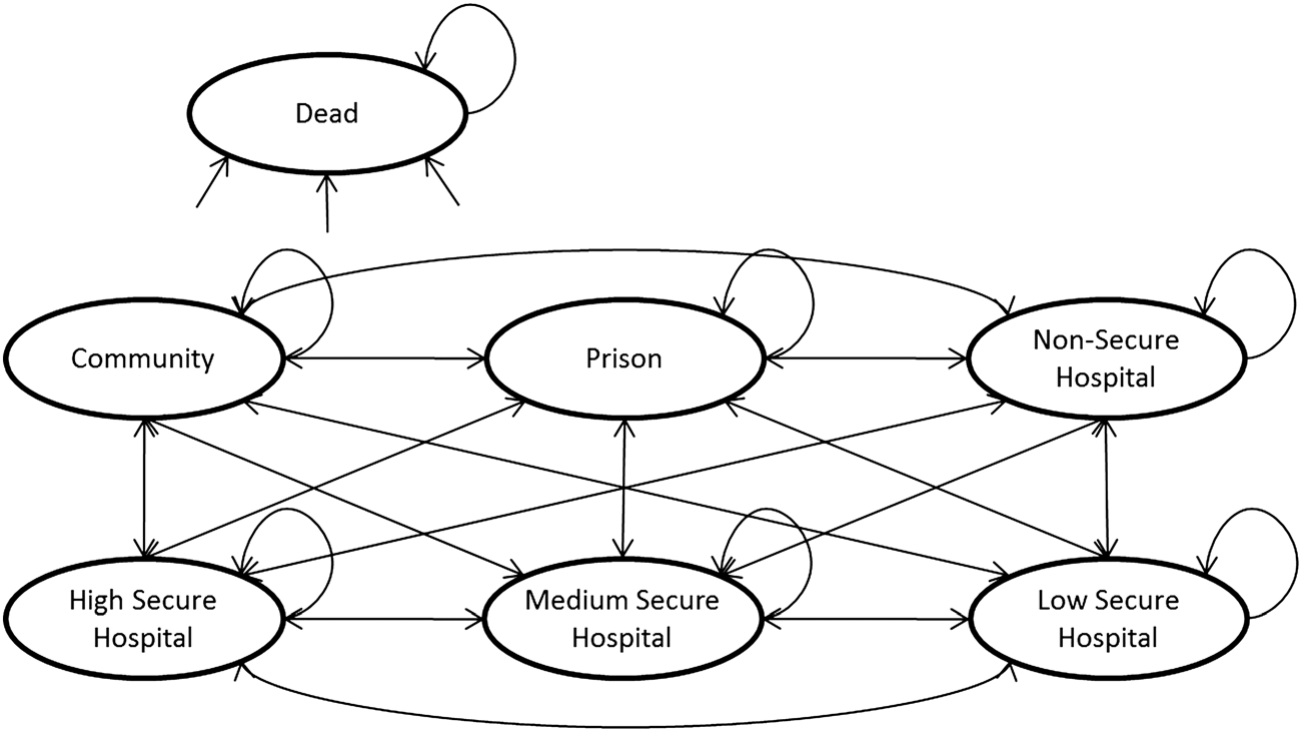
Markov model

Major costs of treatment of offenders with personality disorder follow from the location of the individual. Individuals may reside in the community, prison or hospital. Hospitals may be non-secure or high, medium or low secure. Including the state of being dead, this gives seven possible independent Markov states, as shown in Figure 1. The focus of our study is on costs, so specific outcome measures are not included. The aim of personality disorder services, however, is to rehabilitate people in order to return them safely to a community setting, so location, as defined by Markov states, may be considered an outcome in this respect.

Individuals in our model may move in any direction between any of the six living states, as well as remain in them for successive periods. ‘Dead’ is an absorbing state, transitions to which can be made from any other state, but from which no transitions can be made. Our model depends on the inclusion of two types of parameter: transition probabilities – the probability of moving from one state to another – and state costs. Other types of parameters, such as the quality of life associated with different states or the costs associated with state changes, might have been included but were not for the purpose of this analysis. Our transition probability and cost parameters were obtained from a number of sources, as described later.

Following discharge from a secure unit, individuals with personality disorder may move between states very frequently, so a Markov cycle of more than a month would be insensitive to changes in costs and outcomes and would be misrepresentative of reality. We implemented a model with a weekly Markov cycle. As we are interested in the longer-term consequences of non-completion, we used a 10-year time horizon for our model. Our model is divided into two arms: one for those who complete treatment and one for those who do not. Individuals are assumed not to die during the pre-discharge stage, as this would render them neither a completer nor a non-completer. After discharge, each arm of our model enters separate Markov simulations of post-discharge activity, with identical states and costs but different transition probabilities. Simulations begin from the point at which an individual is first admitted to hospital. We used time-to-event analysis to estimate the probability of discharge in a given Markov cycle. Statistical and regression analyses were carried out using Stata 12 (Stata Statistical Software, College Station, Texas, USA), and the Markov model was constructed using Microsoft Excel 2010 (Redmond, Washington, USA). The complete model Excel file is available in the supplementary online material.

### Data

Our analysis used data from a specialist National Health Service (NHS) service at Arnold Lodge, a medium-security hospital facility in Leicester, England. This service provides treatment for offenders with a diagnosis of personality disorder and offers structured cognitive behavioural interventions alongside a therapeutic community approach modified for the secure setting (McMurran et al., 2001; McCarthy and Duggan, 2010). The sample was of 95 patients, each of whom met diagnostic criteria for personality disorder according to the International Personality Disorder Examination (Loranger, 1994). Data were collected between 1999 and 2009, with a follow-up period of up to 10 years after discharge.

A clinical judgement on completion status was made at the time of discharge for purposes separate from this study. We used this information to separate our sample into two subgroups: ‘completers’ (*n* = 24) and ‘non-completers’ (*n* = 71). The former group comprised those who completed treatment and the subsequent planned discharge. Members of the non-completer group had been discharged early because of poor behaviour (for example, verbal aggression, violence or drug use) or premature disengagement or were unable to engage in the treatment because of other previously undetected mental health problems.

#### Transitions

The transitional probabilities of our model are used to determine which Markov states individuals are likely to move from and to in any Markov cycle. These probabilities were calculated by eliciting the rate of each possible transition between the community, prison and hospital states in every week of the observed 10-year follow-up. These rates were then averaged and converted to weekly probabilities. Transition probabilities were calculated separately for treatment completers and non-completers. Using this method, we were able to estimate the standard errors for transition rates, allowing us to incorporate a probabilistic sensitivity analysis as discussed in succeeding text. As stated earlier, all transitions are possible in practice; however, because of the size of our sample, some transitions were not observed. Our model assumes that, in all states, individuals face an equivalent risk of death. A recent study of post-discharge outcomes from Arnold Lodge found that individuals with personality disorder had a fourfold greater risk of dying than the general population (Davies et al., 2007), but the Davies study was based on a small and potentially high-risk group. In their study of mortality rates for those with mental disorder, Harris and Barraclough (1998) found that individuals with personality disorder have, on average, a standardised mortality ratio of 1.84. As such, we chose to use age-specific and gender-specific rates from English standard life tables, inflated by 84%. A sensitivity analysis was carried out to test the effect on our results of using standard mortality rates. Discharge locations and length of stays were known for each individual. Using these data, we estimated a time-to-event model, where the event of interest was discharge from Arnold Lodge, dependent on an individual's completion status. We implemented a parametric Weibull regression model, which enabled us to obtain time-dependent discharge probabilities that increased or decreased over time, as appropriate, depending on completion status.

#### Costs

Our main aim was to demonstrate how, for a given population of forensic inpatients with personality disorder, non-completion of treatment may lead to differing costs after discharge. The sole focus of this model is therefore upon costs, from a combined perspective of the NHS and criminal justice system. Costs were assigned using a number of different sources and attached to resource use. NHS Reference Costs (Department of Health, 2011) were used for the cost-per-bed of hospital stays. Our figure for the average cost per prisoner is taken from a recent report by the Prison Reform Trust (2009). An accurate measurement of the cost of individuals in the community was less readily available, and we have chosen to use that found by a 2002 study of the economic impact of personality disorders in the UK (Rendu et al., 2002) (inflated to 2010 prices). We assumed there to be no cost associated with an individual being dead. The mean cost of one Markov cycle in each state, and its standard error, is shown in Table 1. To take into account the diminished present value of future costs, and in line with UK National Institute for Health and Clinical Excellence guidelines (NICE, 2009), costs were discounted at an annual rate of 3.5% with model progression. Pre-discharge costs were estimated using the figure for a medium secure stay shown in Table 1, and these were applied to an individual's length of stay at Arnold Lodge before entering the Markov model.

**Table 1:** Weekly Markov state costs

	Community	Prison	Low secure	Medium secure	High secure	Non-secure	Dead
Mean	£12.09	£865.38	£2926.97	£3366.07	£5352.55	£2128.43	£0
*SE*	1.21	—	293.19	516.00	743.28	166.75	—

### Analysis

Key outputs of the model include the cost incurred by completers and non-completers, on average, after 10 years from admission. In addition to this, we present results in terms of cohort state distributions; the proportion of individuals residing in different states. We also present the results from a number of sensitivity analyses. It was important, in the case of our study, to take into account the considerable uncertainty around our parameters. We therefore, as far as possible, implemented a probabilistic sensitivity analysis (Doubilet et al., 1985). In the case of transition probabilities between community, prison and hospital states, we used standard errors to sample from a distribution of values. For probabilities, we use a beta distribution, which is bound by 0 and 1, as is customary (Briggs et al., 2006). Mortality rates were not directly made probabilistic but age was, with a normal distribution assumed. For costs relating to hospital stays, we used Excel's Solver (Frontline Systems, Inc., Incline Village, Nevada, USA) function to fit an estimated standard error of the unit cost from the lower-quartile and upper-quartile values provided in the reference costs. As is recommended (Briggs et al., 2006), we assumed a gamma distribution and sampled probabilistically using our estimated standard errors. The only cost for which we were not able to sample from a distribution was the cost of prison. Our Weibull time-to-event estimations for length of stay were made probabilistic using the Cholesky decomposition method.

Our model implemented 10,000 simulations, as we wanted to be sure to take account of the high level of uncertainty in our data. Each simulation samples a random value from the parameter's assumed distribution. By making our model probabilistic, our results are less likely to be skewed by outliers and extreme observations. Further sensitivity analyses were carried out on specific parameters. We investigated the effect of allowing transitions to occur that were not observed in the data, but that we would expect to occur occasionally in reality. Under ideal circumstances it would have been possible for us to carry out a cost-effectiveness analysis, though without data on health states or other specific outcomes this was not possible.

In addition to our primary results, we present results of a post-discharge model. We believe this to be a relevant perspective for practitioners and commissioners, as there is likely to be on-going provision of specialist hospital-based treatment for high-risk personality disordered offenders. This perspective may inform existing services focused on enhancing completion.

## Results

### Sample characteristics

The mean follow-up period was over 5 years. Clinical and demographic information is summarised in Table 2. All the patients were men. The mean age at discharge was 29 years (median 28, range 18–45). The treatment completion rate was 25%; slightly lower than that found in a review of previous studies (McMurran et al., 2010). Mean length of stay for treatment completers was 97 weeks (s.d. = 41, range 30–216), compared with 28 weeks (s.d. = 27, range 1–126) for the non-completers. On the five-point Tyrer and Johnson (1996) scheme, 23% of the sample was classified as having severe or very severe personality disorder (4, 17% of treatment completers; 18, 25% non-completers). Most men in each group had personality disorders in only one cluster. The sequential post-discharge destinations were known for each man, as was the subsequent number of weeks he spent in prison, hospital or community in each follow-up year. The data provided over 20,000 weekly locations for the sample. In order to ensure representation of reality, we chose not to use any exclusion criteria in this study, aside from death during initial inpatient treatment (which was not observed).

**Table 2:** Admission characteristics of the sample

		Non-completers (*n* = 71)	Completers (*n* = 24)	*P*-value^b^	Whole sample (*n* = 95)
Personality disorder severity (5-way) (Tyrer and Johnson, 1996)^a^	0	4 (6%)	2 (8%)		6 (6%)
	1	43 (61%)	15 (63%)		58 (61%)
	2	6 (8%)	3 (13%)	0.522^c^	9 (9%)
	3	10 (14%)	2 (8%)		12 (13%)
	4	8 (11%)	2 (8%)		10 (11%)
White		67 (94%)	21 (88%)	0.509	88 (93%)
Married		3 (4%)	2 (8%)	0.802	5 (5%)
Admitted from prison or court		54 (76%)	17 (71%)	0.812	71 (75%)
Education: GCSE or above		25 (35%)	6 (25%)	0.503	31 (33%)
Previous mental health inpatient treatment		25 (35%)	13 (54%)	0.162	38 (40%)
Previous significant alcohol misuse		47 (66%)	16 (67%)	0.835	63 (66%)
Previous significant drug misuse		53 (75%)	12 (50%)	0.046	65 (68%)

GCSE = General Certificate of Secondary Education.

a 0 = PD not otherwise specified; 1 = simple, PD in one cluster; 2 = diffuse, PD in two or more clusters; 3 = severe, anti-social PD plus at least one PD in cluster A or cluster C; 4 = very severe, as for ‘severe’ but scoring 25 or greater on the Revised Hare Psychopathy Checklist.

b Chi-squared test.

c Wilcoxon–Mann–Whitney test.

### Probabilistic results

On the basis of 10,000 iterations of our model, over the first 10 years after admission, the average cost was £499,759 for completers and £551,473 for non-completers; a mean cost difference of £51,714. The results from our probabilistic model show that there is a 78% chance that, on average, treatment completers incur lower costs than non-completers in the first 10 years following admission to Arnold Lodge. The primary driver of this cost differential is that non-completers tend to be discharged to prison, whereas completers tend to be discharged to the community. Furthermore, non-completers are likely to spend more time in hospital because of readmissions following discharge.

#### Post-discharge simulation

If our model is simulated for 10 years from the point of discharge, a more substantial and significant cost difference is apparent. The average 10-year follow-up cost for completers was £266,396 and for non-completers £410,526, a mean cost difference of £144,130. In this case, we found there to be a 97% chance that treatment completers, on average, go on to incur lower costs than non-completers in the first 10 years following discharge.

### Deterministic results

The cumulative costs incurred by completers and non-completers in the 10 years following admission are shown in Table 3. These deterministic results demonstrate that the cumulative costs in both groups increase, but at a decreasing rate. Annual costs for completers, however, decrease more quickly. The greater post-discharge costs incurred by non-completers lead to an overall cost differential by the seventh year after admission.

**Table 3:** Mean cumulative post-admission costs

Years	Completers	Non-completers	Difference
1	£147,203	£114,968	£32,235
2	£240,233	£178,432	£61,801
3	£298,225	£233,887	£64,338
4	£337,892	£285,516	£52,375
5	£369,047	£334,009	£35,038
6	£396,579	£379,922	£16,657
7	£422,662	£423,666	£−1004
8	£448,178	£465,527	£−17,349
9	£473,444	£505,696	£−32,252
10	£498,547	£544,300	£−45,753

#### Cohort distributions

The model also shows differences in where treatment completers and non-completers tend to go after discharge. Table 4 shows the distribution of individuals in different states at the end of each of the first 5 years following discharge, with the four post-discharge hospital states collapsed into a single figure. In each of the first 5 years, the proportion of completers in the community is greater than that for non-completers. Annual differences in proportions of completers and non-completers in prison in each year are small and relatively constant.

**Table 4:** Post-discharge cohort state distributions

		Completers (%)	Non-completers (%)
Year 1	Community	56	49
	Prison	38	40
	Hospital	5	11
Year 2	Community	55	45
	Prison	39	40
	Hospital	6	15
Year 3	Community	54	42
	Prison	39	40
	Hospital	7	18
Year 4	Community	53	41
	Prison	39	40
	Hospital	8	19
Year 5	Community	52	40
	Prison	38	39
	Hospital	9	20

### Sensitivity analyses

If we extend our model by a further 10 years, to 20 years post-admission, the cost difference is more pronounced. Completers incur an average cost of £735,061, whereas non-completers incur £874,536, a difference of £139,475. In this case, the probability that non-completers cost more is 83%. As our sample was rather small, we found that some transitions were never made. We expect that, in reality, these transitions would have very low positive values. As such, we adjusted these transition probabilities to be non-zero for the sake of sensitivity analysis. The result of increasing all of these weekly probabilities to 0.01% did not remove the average 10 year cost differential between completers and non-completers, although it was reduced to £25,278, and the probability that completers incurred less cost reduced to 65%. Standardising mortality rates to match that of the population of England causes a negligible increase in costs across both groups.

## Discussion

### Main findings

Using methods of decision-analytic modelling, we were able to identify the expected difference in costs incurred by treatment completing and non-completing men with personality disorder, in the first 10 years following admission, and to estimate the probability that treatment completers go on to be less costly than non-completers overall. Our study was the first to evaluate the economic consequences of non-completion of treatment for personality disorder, and one of very few that have used modelling techniques.

Our analyses also show that the estimated cost difference is likely to hold when the greater cost of initial treatment for the completers is taken into account. It seems likely that the reason for lower longer-term costs is that treatment completers, on average, spend more time in the community, and less time in prison or hospital, than non-completers. In the years following discharge, there is an increasing proportion of non-completers residing in one of the three hospital states. These results are consistent with findings of previous studies (Webb and McMurran, 2009; McCarthy and Duggan, 2010).

It is important to note that our results do not demonstrate any causal relationship between treatment completion and post-discharge costs, but rather one of correlation. We hope that future research will investigate whether a causal relationship does exist. Nonetheless, these results do offer an incentive to ensure that a greater proportion of individuals complete treatment. An increase in completion rates might be achieved through staff training in engagement techniques and interventions to improve treatment readiness. A recent meta-analysis found that interventions to increase attendance for psychotherapy have been found to be moderately effective (Oldham et al., 2012). As such, there may be further benefits to increasing completion rates beyond cost savings. Alternatively, savings might be made, and outcomes improved, by refining the process of selection into treatment. If a causal link exists between non-completion and higher costs, we would expect an increase in the completion rate to save money. On the basis of our estimations, an increase in the completion rate from 25% to 50% would save an average of more than £,1000 per person per year. For a cohort of 100 individuals, this represents a saving of £1.1m over a 10-year period.

### Strengths and limitations

Our study benefits from the quality of data used. In the field of personality disorder, particularly in relation to treatment completion, studies tend to be based on very limited data with small populations and incomplete follow-up. In their review of the correlates and consequences of non-completion in treatment for personality disorder, for example, McMurran et al. (2010) found a median sample size of 60 in relevant studies. Furthermore, the data used in our study have been used for other purposes elsewhere, and there were few missing items. These data have been found to provide robust, consistent and intuitive results in other studies (Davies et al., 2007; McCarthy and Duggan, 2010), but have never before been used in an economic analysis. In decision-analytic modelling, it is often difficult to obtain data from which regular transition probabilities can be elicited. Our data provided enough information for us to implement weekly iterations of our model. The length of follow-up data made available to us (up to 10 years) further reinforced our analysis by reducing the uncertainty in our long-term simulations. Our chosen method of analysis also enabled us to take the uncertainty in our parameters into account more fully than traditional hypothesis testing methods allow.

There are also, however, a number of limitations to our study. First, our data are taken from a medium secure forensic unit, and our results may therefore not be generalisable to other forensic populations. Equally it could also be argued that our data are not specific enough to our population as, for example, prison costs used in the model are not specific to those with personality disorder. It has been estimated, however, that 61% of prisoners are likely to have a diagnosable personality disorder (Stewart, 2008), so the general prison population may be more similar than not to these men. Second, the range and quality of the parameters we included would need improvement for an analysis from a societal perspective. Our individual costs, for example, were heavily underestimated as they did not take into account costs of crime and criminal justice aside from imprisonment. Given our knowledge of individuals' discharge locations, however, and that studies have shown that treatment non-completion is associated with higher rates of reoffending and reconviction (McMurran and Theodosi, 2007), we would thus expect the real cost differences to be substantially higher than our estimates. Similarly, the costs associated with discharge and hospital transfers were not taken into account. Third, our study makes a number of assumptions, as described earlier. One important assumption of our model is that transition probabilities remain constant over time, which is almost certainly not the case, but our data were not sufficient to enable us to elicit time-variant transition probabilities.

There are a number of questions that this study leaves unanswered. Our study only compares treatment completion with treatment non-completion. Ideally, we would have liked our analysis to incorporate a no treatment arm, but appropriate data were not available. We hope that in the future our model can be used for such an analysis as this is crucial in identifying an optimal policy. It is possible that the difference in costs between the groups is due to heterogeneity. It may be the case that treatment completers and non-completers differ in ways that make them more or less likely to incur costs after discharge, for reasons other than their completion status. Some research has shown that there are underlying differences between treatment completers and non-completers (McMurran et al., 2008). A recent study using the same dataset, however, demonstrated that, in terms of their psychometric properties, completers and non-completers do share a number of characteristics (McCarthy and Duggan, 2010). It is therefore crucial that future research investigates the underlying determinants of non-completers' higher costs and thus the true isolated cost impact of non-completion. It is also possible that our chosen indicator for ‘completion’ is not appropriate and may not be robust across other populations. Further research is necessary to establish a consistent indicator of personality disorder treatment completion. A complete decision-analytic model would ideally take quality of life measures into account too, which was not possible here.

### Implications

Our findings suggest that it is likely that an improvement in treatment completion rates for men with personality disorder would lead to some cost savings. It is possible that interventions designed to increase the completion rate through better patient engagement or staff training would both enhance completion and reduce overall costs. Alternatively, an improvement in selection procedures might have a similar effect on long-term overall costs. In addition, we have demonstrated the value of decision-analytic modelling in this area of study, where comprehensive sets of data are rare. We hope future research will be facilitated by routine monitoring of treatment completion in secure hospital units.
